# Understanding the Sequence-Dependence of DNA Groove Dimensions: Implications for DNA Interactions

**DOI:** 10.1371/journal.pone.0015931

**Published:** 2010-12-29

**Authors:** Christophe Oguey, Nicolas Foloppe, Brigitte Hartmann

**Affiliations:** 1 Laboratoire de Physique Théorique et Modélisation, UMR-8089, Centre National de la Recherche Scientifique et Université de Cergy-Pontoise, Cergy-Pontoise, France; 2 UMR-S665, Institut National de la Santé et de la Recherche Médicale et Université Paris Diderot, Institut National de la Transfusion Sanguine, Paris, France; New England Biolabs, Inc., United States of America

## Abstract

**Background:**

The B-DNA major and minor groove dimensions are crucial for DNA-protein interactions. It has long been thought that the groove dimensions depend on the DNA sequence, however this relationship has remained elusive. Here, our aim is to elucidate how the DNA sequence intrinsically shapes the grooves.

**Methodology/Principal Findings:**

The present study is based on the analysis of datasets of free and protein-bound DNA crystal structures, and from a compilation of NMR ^31^P chemical shifts measured on free DNA in solution on a broad range of representative sequences. The ^31^P chemical shifts can be interpreted in terms of the BI↔BII backbone conformations and dynamics. The grooves width and depth of free and protein-bound DNA are found to be clearly related to the BI/BII backbone conformational states. The DNA propensity to undergo BI↔BII backbone transitions is highly sequence-dependent and can be quantified at the dinucleotide level. This dual relationship, between DNA sequence and backbone behavior on one hand, and backbone behavior and groove dimensions on the other hand, allows to decipher the link between DNA sequence and groove dimensions. It also firmly establishes that proteins take advantage of the intrinsic DNA groove properties.

**Conclusions/Significance:**

The study provides a general framework explaining how the DNA sequence shapes the groove dimensions in free and protein-bound DNA, with far-reaching implications for DNA-protein indirect readout in both specific and non specific interactions.

## Introduction

The cellular DNA is continuously “read” by proteins. DNA-protein interactions are informed by the intrinsic mechanical properties of DNA, which facilitate its deformation in the complex. The DNA binding process depends on the intrinsic ability of free DNA to adopt its structure when bound to a protein. Therefore, understanding the origins of the conformational preferences of the free nucleotidic DNA sequences remains an important goal in structural biology.

In DNA-protein complexes, proteins fit snugly in the DNA major and minor grooves. Thus, sequence specific variations of the DNA grooves play a central role in DNA-protein readout processes [1]–[7]. The major groove dimensions remain quite similar in free and bound DNA; in contrast, the minor groove is especially variable in complexes [3], [6]. Indeed, the increasing number of X-ray structures highlighted the importance of DNA minor groove in DNA-protein complexes. More contacts than expected are observed in the DNA minor groove [8]. Architectural proteins [1] and proteins binding DNA sequences non-specifically [9] mainly interact with the DNA minor groove, where there is little discrimination between base types [10]. Nevertheless, this type of interaction is still intriguing since the DNA minor groove is often presumed too narrow to accommodate protein structural elements without energetically costly distortions.

This view has been recently revisited [6] in a study addressing very large datasets of free and bound X-ray DNA structures, showing that the electrostatic potential in the minor groove is influenced by its geometry, and can be recognized by proteins. Poisson-Boltzmann calculations revealed that narrow minor grooves exhibit an enhanced negative electrostatic potential, favoring their interaction with arginine residues. This electrostatic effect is particularly marked in A•T rich segments that tend to adopt very narrow minor grooves in both free and bound DNA crystal structures. This interesting finding helps to understand how proteins can penetrate into a narrow DNA minor groove but does not explain the mechanisms underpinning the enlargement of the minor groove in many bound DNAs, which can be considerable [3], [6]. Thus, understanding the origin of minor groove widening remains a key question in structural biology, with far-reaching implications for DNA readout.

**Figure 1:**
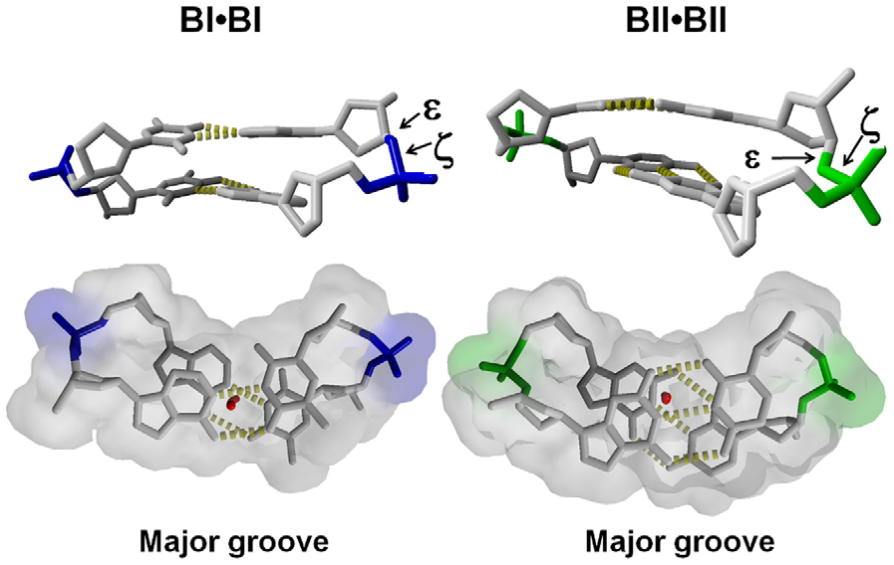
Illustration of the BI and BII phosphate linkages, and associated shift of the base-pairs relative to the helical axis. The phosphate groups in BI (in blue, left panels) and BII (in green, right panels) conformations differ in the torsion angles ε and ζ, respectively *trans/g-* in BI and *g-/trans* in BII. Compared to the BI steps, BII steps shift the base-pairs towards the major groove (bottom panels), as shown by reference to the trace of the helical axis in red. The BI•BI ApA•TpT (left) and BII•BII GpG•CpC (right) steps are from the PDB structures 1EHV and 3GGI, respectively.

Crystallographic analyses of DNA-protein complexes suggested that conformational sub-states of the DNA phosphodiester backbone may be implicated in the minor groove binding mechanisms [8], [11], [12]. Hydrophobic contacts in the DNA minor groove could be aided by *south* to *north* sugar switches, *north* sugars increasing the accessibility of both sugars and bases [12]. Also, the BI and BII conformational sub-states of phosphate groups appear associated with minor groove modulations in several DNA-protein complexes [8], [11], [13], [14]. These conformers were identified from crystallographic studies [15] and by NMR [16], [17]. They are defined by the torsion angles ε and ζ, *trans/g-* in BI (ε-ζ∼−90°) and *g-/trans* in BII (ε-ζ∼+90°) (Figure 1). Binding of the DNA minor groove by amino-acids is often accompanied by changes in ε and ζ [8], [11]. The DNA of Nucleosome Core Particles alternates narrow and wide minor grooves associated with positive and negative rolls [18], in turn coupled with BI- and BII-rich regions [14], [19], respectively. The relationship between the DNA backbone properties and protein binding *via* the minor groove was further demonstrated by probing the indirect readout mechanism underlying the DNA-DNase I interaction [13]. The DNase I amino acids fill the DNA minor groove, which becomes much wider than in canonical B-DNA [20]. Exploiting exhaustive NMR studies [21], [22] showed that the intrinsic BII propensities characteristic of free DNA correlate with the differential DNase I cleavage intensities [13]. Indeed, the NMR-refined free DNA structures revealed that BII-rich phosphates favor wide minor grooves, promoting the affinity for DNase I and thus the DNase I cleavage efficiency [13]. These experimental observations correlating BII populations and minor groove geometry parallel a molecular modeling investigation, which also suggested that minor groove opening in free DNA is associated to a larger proportion of BII backbones [23]. Overall, these particular systems indicate that the minor groove shape is strongly influenced by the corresponding BI/BII propensities, and it would be of great interest if one could explain and generalize these effects.

More generally, there is growing evidence that the DNA backbone BI↔BII equilibrium strongly impacts indirect DNA readout by proteins [13], [14], [17], [19], [21], [22], [24]–[27] for two main reasons: i) the BI↔BII equilibrium is sequence-dependent [13], [14], [28], [29] and ii) the BI/BII ratios are closely related to the overall shape of B-DNA [21], [28], [30]–[33].

The BII propensities are primarily controlled at the dinucleotide level, as shown from X-ray [14], [28] and NMR data [19], [29]. In solution, the BI/BII ratios are inferred from the ^31^P chemical shifts [29], [34], very accurately measured by NMR. Each of the 16 B-DNA dinucleotides is characterized by a specific ^31^P chemical shift average value, and thus by a specific BII percentage [19]. From a structural point of view, the BI↔BII equilibrium is coupled to the deoxyribose conformational exchange [14], [28], [35] and to the DNA helicoidal parameters of twist, roll (correlated to slide) and base-pair displacement (X-disp) [21], [28], [30]–[33]. The variations in B-DNA structures described by other helicoidal parameters, for instance rise or tilt, are too weak [36] to result in significant correlations with the backbone states (or other structural parameters). Thus, the BI/BII ratio reflects the most variable DNA helicoidal descriptors.

These DNA properties were recently exploited to conceive the so-called TRX scale, which quantifies the intrinsic flexibility of the dinucleotides in free B-DNA, which varies greatly [19]. In this scale, each complementary dinucleotide step is characterized by a numeric score (the experimental average BII population of its facing phosphates), reflecting the flexibility of the relevant phosphates. Due to the tight coupling between helical parameters and backbone states, this number also represents the helicoidal malleability in terms of Twist, Roll and X-disp. According to this scale, ApA•TpT, ApT•ApT, TpA•TpA, ApC•GpT and ApG•CpT are categorized as stiff steps. In contrast, GpG•CpC, GpC•GpC, CpG•CpG and CpA•TpG have an enhanced flexibility. GpA•TpC appears intermediate, with GpA flexible, but not TpC. Quantifying the overall flexibility of each of the ten complementary dinucleotide steps, the TRX scale provides fresh insights to understand how the DNA/protein interactions depend on DNA's intrinsic, sequence-dependent, malleability.

The helical parameters, influenced by the BII propensities, are connected to groove dimensions [5], [7], [37], [38]. For example, the base-pair displacements are directly related to the groove depths; positive and negative rolls compress the minor and major grooves, respectively. Here, we explain the structural coupling between the backbone states and the groove dimensions. In particular, comparing groove geometries from high resolution X-ray DNA structures to the independently derived TRX scale provides a general explanation of the minor groove width variations. Then, exploiting a very large dataset of DNA/protein complexes recently presented [6], we show that proteins recognize the intrinsic sequence-specific malleability of DNA grooves. This offers a new and powerful explanatory insight on how proteins read DNA.

## Results

### Relationship between groove dimensions and phosphate group conformations

In B-DNA, the phosphate groups adopt two conformations, BI (ε-ζ∼−90°) and BII (ε-ζ∼+90°) (Figure 1). Crucially, the B-DNA intrinsic mechanics involves a tight relationship between the backbone conformations and the inter base-pair rotational parameters of roll and twist [21], [28], [30]–[33], [39]. In addition, BI phosphate groups are associated to base-pairs positioned at the center of the double helix, while BII linkages are accompanied by base-pairs displaced off-center, towards the major groove (Figure 1). This displacement, modulated by the neighboring base-pairs to minimize stacking discontinuity [31], [33], is especially pronounced when several proximal phosphates are in BII [28], [30], [32]. This base-pair displacement is directly related to groove depth, a high density of BII steps leading to shallow major grooves and deep minor grooves.

Here, we investigate the relationship between the phosphate group conformations and the groove dimensions, including the major and minor groove widths. Our analysis was based on high resolution X-ray structures of DNA decamers (list provided in Supporting Information S1), without including the dodecamers engaged in contacts with adjacent replicas *via* their grooves, which could be artificially distorted [40]–[42]. We considered the phosphate conformations within pNpNp•pNpNp segments (N: any nucleotide) instead of NpN•NpN, since the displacement of a given base-pair N•N depends on its flanking phosphate conformations (pNp•pNp). The observed combinations of phosphate group conformations in pNpNp•pNpNp are summarized in Table 1. Pairs of BII phosphates adjacent on a strand (BII-BII repetition) are very rare (2 cases out of 139). Therefore, BII-rich regions typically involve BII and BI phosphates alternating on a same strand, each BII phosphate being surrounded by two BI steps [14], [28], [35].

**Table 1:** Effect of BII phosphate groups on DNA groove dimensions.

		Minor groove		Major groove	
n	BI/BII configurations	Width	Depth	Width	Depth
65	5′-pNpNp-3′•5′-pNpNp-3′	4.6 (1.4)	5.3 (0.4)	11.8 (1.1)	4.2 (1.4)
27	5′-pNpNp-3′•5′-pNpNp-3′	5.9 (0.9)	5.6 (0.5)	10.6 (1.1)	3.8 (1.2)
4	5′-pNpNp-3′•5′-pNpNp-3′	5.7 (1.0)	5.6 (0.5)	11.9 (1.4)	3.4 (1.1)
2	5′-pNpNp-3′•5′-pNpNp-3′	6.1 (0.7)	5.9 (0.2)	11.6 (0.4)	3.5 (0.6)
5	5′-pNpNp-3′•5′-pNpNp-3′	6.4 (1.1)	5.6 (0.8)	10.9 (1.1)	3.8 (0.7)
13	5′-pNpNp-3′•5′-pNpNp-3′	6.3 (0.5)	5.5 (0.5)	11.0 (0.6)	3.0 (0.4)
2	5′-pNpN***p***-3′•5′-pNpNp-3′	7.0 (0.3)	5.3 (0.7)	11.6 (0.5)	3.3 (1.1)
5	5′-pNpNp-3′•5′-pNpNp-3′	7.5 (0.4)	5.0 (0.5)	11.4 (0.7)	5.4 (0.3)
14	5′-pNpNp-3′•5′-pNpNp-3′	7.5 (0.4)	6.1 (0.5)	10.6 (0.7)	2.8 (0.5)
2	5′-pNpNp-3′•5′-pNpNp-3′	7.6 (0.1)	6.5 (0.1)	9.8 (0.1)	3.2 (0.2)

The groove dimensions (in Å, standard deviations in parentheses) were measured on 139 complementary dinucleotides NpN•NpN (N: any nucleotide) in 28 high resolution X-ray structures of free decamers (list provided in Supporting Information S1). The dinucleotides are categorized according to the conformational states of their central and 3′/5′-phosphate linkages. BII phosphates are boxed. The number of occurrences in each category is given by n. The standard deviation for n = 2 is still reported, since the groove dimensions are measured at four points along one dinucleotide step [46].

**Figure 2:**
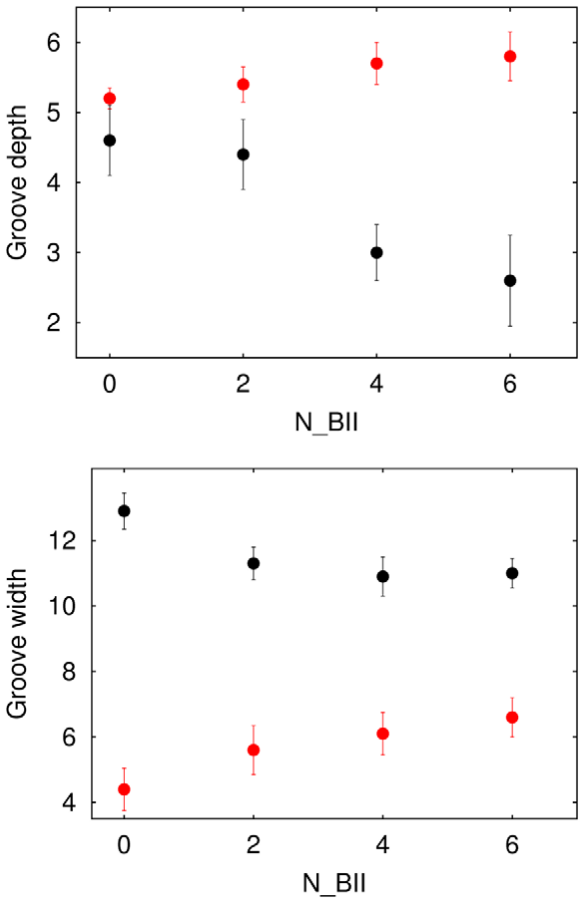
Cumulative BII phosphate groups and DNA grooves. The minor (red) and major (black) groove depths (top) and widths (bottom) (Å) were averaged and categorized according to the number of BII linkages (N_BII) in the 12 central phosphate groups (6 per strand) of 32 high resolution X-ray decamer structures (list provided in Supporting Information S1). The vertical bars correspond to the standard deviations.

Table 1 and Figure 2 highlight that major and minor groove dimensions are extremely sensitive to the conformation of the central and 3′-phosphates in the considered DNA segments. This is particularly apparent for the major groove depth and the minor groove width, which display the most striking variations (∼3 Å between their minimal and maximal values) among the four parameters describing the grooves. As already proposed [21], [28], [30], [32], [33], [39], the major groove becomes shallow in presence of BII, especially with one or two face-to-face BII (major groove depth ≤3.3 Å, Table 1). Major and minor groove depths being anti-correlated (Figure provided in Supporting Information S1), BII-rich segments are also associated to an increased minor groove depth. This relation between groove depth and backbone states is a direct manifestation of the coupling between the BI and BII conformers and the base-pair displacement.

Also, the phosphate conformations strongly influence groove width values. An increasing number of BII states narrows the major groove and broadens the minor groove, although minor and major groove widths are poorly anti-correlated (Figure provided in Supporting Information S1). The widening of the minor groove is especially remarkable: it widens by 3 Å in NpN•NpN surrounded by three or four BII phosphates, compared to purely BI pNpNp•pNpNp tracts (Table 1). This minor groove opening is mechanically associated to the accumulation of negative rolls in BII-rich segments [21], [28], [30]. We recall that the roll angle measures the rotation between two successive base-pair planes about their long axis (y-axis); the roll is negative when it opens up on the major groove side of the bases.

**Figure 3:**
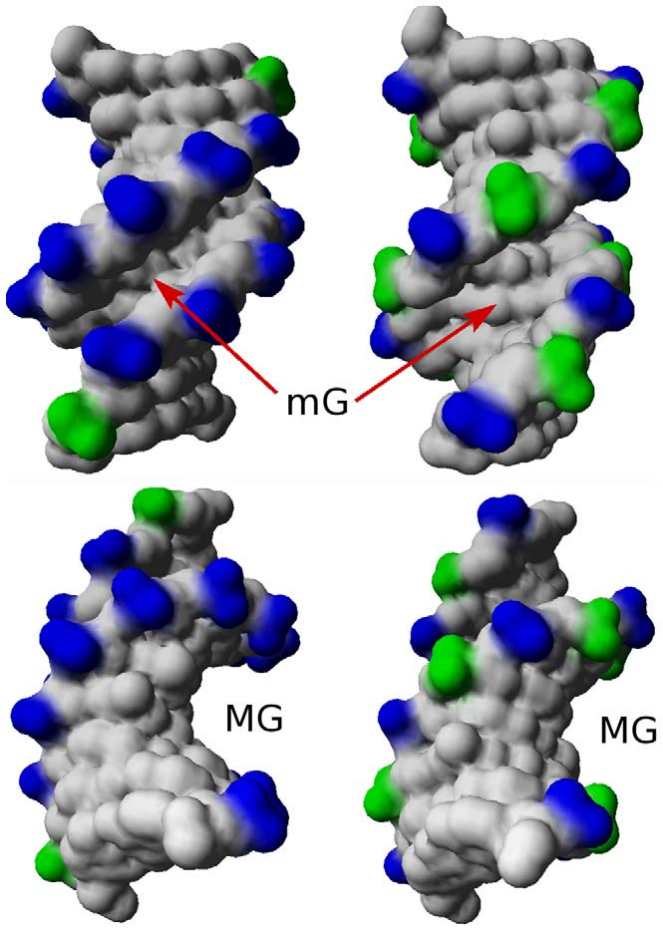
Illustration of the relationship between BI- or BII-rich regions and DNA grooves. The BI-rich (PDB code 1EHV, left) and BII-rich (PDB code 3GGI, right) decamer X-ray structures are in the same orientations. BI and BII phosphate groups are in blue and green, respectively. Top: the lateral view shows that the minor groove (mG) is considerably enlarged in the BII-rich structure (right). Bottom: view along the major groove (MG), showing its reduced concavity in the BII-rich structure (right).

In any decamer, the groove dimensions are defined over the 6 central base-pairs. Considering these central regions and their 3′ neighbors, (Np)_6_•(Np)_6_, confirms the cumulative effect of BII phosphates on B-DNA groove dimensions, modulated by the density of BI or BII phosphate groups (Figure 2). The differential effects of BI- or BII-rich regions on the groove dimensions are illustrated on two representative decamers in Figure 3, clearly showing a wider minor groove and a shallower major groove in the BII-rich decamer.

The mechanism relating phosphate group behavior to groove dimensions is particularly interesting in regard to the minor groove width variations. Because the BI/BII propensities at a given phosphate are characteristic of the corresponding DNA dinucleotidic sequence [19], one expects the minor groove width to follow a similar pattern if it is mechanically associated to the BI/BII configurations. The next section examines this point.

### Sequence-dependence of minor groove width and DNA flexibility

The sequence-dependent DNA intrinsic flexibility can be characterized by the TRX scale (Table 2), developed from an extensive set of NMR data in solution, collected for each of the 16 dinucleotidic sequences 5′-dNpM-3′ where N and M may be A, T, G, or C [19]. In a double helix, these 16 sequences form ten distinct complementary dinucleotide steps (the 12 non-palindromic sequences reduce to 6 different dinucleotidic DNA fragments; for example, both CpA and TpG correspond to the same CpA•TpG step; the four palindromic sequences GpC, CpG, ApT and TpA give rise to four distinct, symmetric, steps). The TRX scale quantifies the intrinsic flexibility of these ten complementary dinucleotides in terms of BI/BII phosphate populations and helical parameters, twist, roll and X-disp (base-pair displacement). Physically, the steps with high TRX scores (typically GpG•CpC, CpG•CpG, GpC•GpC and CpA•TpG) explore a larger conformational space than those with low TRX scores (typically ApN•NpT).

**Table 2:** Influence of the base sequence on the free B-DNA intrinsic flexibility.

Sequence	%BII	S_TRX_
CpG•CpG	43•43	43
CpA•TpG	52•31	42
GpG•CpC	47•37	42
GpC•GpC	25•25	25
GpA•TpC	33•11	22
TpA•TpA	14•14	14
ApG•CpT	18•0	9
ApA•TpT	11•0	5
ApC•GpT	8•0	4
ApT•ApT	0•0	0

The DNA sequence is expressed in terms of the 10 complementary dinucleotides base steps, as summarized here from a previous study (Heddi et al., 2010b). The intrinsic flexibility of each complementary dinucleotide is quantified by its TRX score (S_TRX_), i.e. the half-sum of the average BII percentages (%BII) observed in solution by NMR for its two facing phosphates. The higher the TRX score, the greater the intrinsic flexibility of the step, especially in terms of the helicoidal parameters twist, roll and X-displacement. The maximal theoretical flexibility corresponds to a TRX score of 50 (50% of time in BI, 50% of time in BII). The average standard deviation of %BII is ±8.

**Figure 4:**
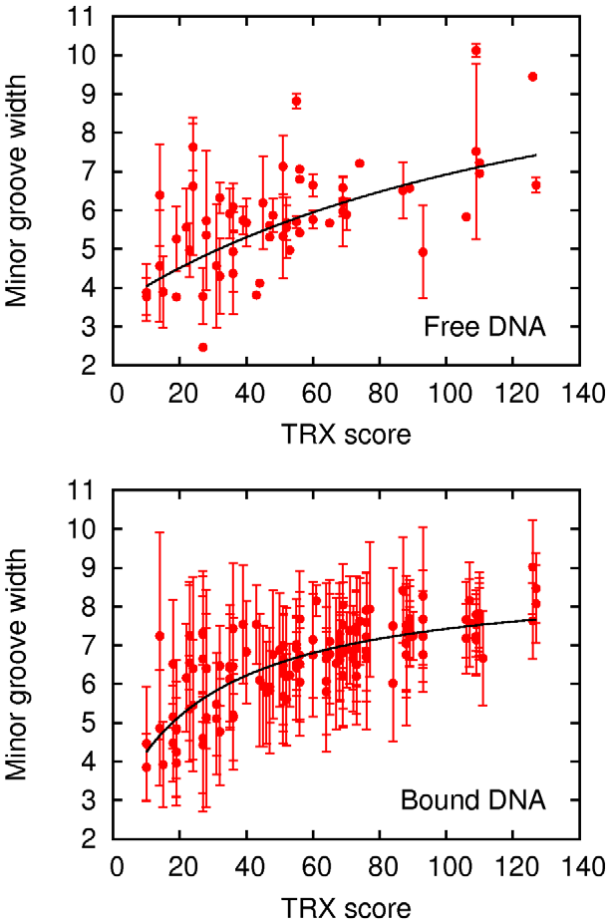
Influence of DNA intrinsic flexibility on the DNA minor groove width. The average minor groove width values (Å) of 59 tetrameric sequences in X-ray structures of free B-DNA (top), and of the 136 possible tetrameric sequences in X-ray structures of protein-bound DNA (bottom) were taken from recently published data [6]. These values are plotted versus the TRX scores calculated on the same DNA sequences, with a sliding tetrameric window. These “tetrameric” TRX scores (Table 3) are the sum of the constituent TRX scores at the base step level (Table 2). The vertical bars correspond to the minor groove width standard deviations. The black curves are non-linear data fits.

Given that the minor groove dimensions measured on dinucleotides depend on the phosphate conformations in pNpNp•pNpNp segments (Table 1), we calculated the TRX score corresponding to a tetrameric window (NpNpNpN•NpNpNpN), as the sum of the individual TRX scores of the three complementary dinucleotides in the tetramer (Table 3). These scores were compared to the exhaustive minor groove width values recently published [6]. These minor groove width data were extracted from a large number of free oligomer X-ray structures, then sorted and averaged according to 59 (out of the 136 possible) tetrameric sequences. Despite a rather large dispersion, likely contributed by crystallographic biases, the tetramer TRX scores broadly reflect the minor groove width values (Figure 4-A). This parallel between the sequence-dependent DNA flexibilities in solution (TRX scores) and the minor groove widths in X-ray structures strongly supports the notion that this relation is a general intrinsic property of DNA. To interpret this relation, one should keep in mind the origins of the quantities, both averaged but extracted from either static (minor groove width values) or dynamic (TRX scores) structures. In particular, high tetrameric TRX scores correspond to flexible tetrameric sequences, in which the phosphate linkages can explore all the BI and BII combinations listed in Table 1. Therefore, malleable sequences oscillate between wide and narrow grooves. The coupling uncovered in Figure 4-A means that higher TRX scores increase the probability of wide minor groove conformations.

**Table 3:** TRX scores of the 136 unique tetranucleotides in free B-DNA.

Tetramer	S_TRX_	Tetramer	S_TRX_	Tetramer	S_TRX_	Tetramer	S_TRX_
AAAT•ATTT	10	AGAA•TTCA	36	GGAT•ATCC	64	CATG•CATG	84
AATT•AATT	10	GAAG•CTTC	36	TGAT•ATCA	64	TCGA•TCGA	87
AAAC•GTTT	14	GATA•TATC	36	CGAT•ATCG	65	GGGT•ACCC	88
ATAT•ATAT	14	AAGC•GCTT	39	TAGG•CCTA	65	GGTG•CACC	88
AAAA•TTTT	15	AGAG•CTCT	40	ATGC•GCAT	67	GTGG•CCAC	88
AAGT•ACTT	18	AGCT•AGCT	43	GGAC•GTCC	68	TGGT•ACCA	88
AGTT•AACT	18	GATC•GATC	44	GGTC•GACC	68	TGTG•CACA	88
ATAC•GTAT	18	TAGA•TCTA	45	GTGA•TCAC	68	ACGG•CCGT	89
AAAG•CTTT	19	ATGT•ACAT	46	TGAC•GTCA	68	CGGT•ACCG	89
AATA•TATT	19	AATG•CATT	47	TGTC•GACA	68	CGTG•CACG	89
ATAA•TTAT	19	CAAT•ATTG	47	ACGA•TCGT	69	TTGG•CCAA	89
TAAT•ATTA	19	TAGC•GCTA	48	CGAC•GTCG	69	GCGA•TCGC	90
GTAC•GTAC	22	GTGT•ACAC	50	CGTC•GACG	69	AGGG•CCCT	93
ATAG•CTAT	23	ACGT•ACGT	51	GGAA•TTCC	69	CAGG•CCTG	93
GTAA•TTAC	23	CAAC•GTTG	51	TGAA•TTCA	69	CTGG•CCAG	93
TAAC•GTTA	23	GGTT•AACC	51	TTGA•TCAA	69	GCGC•GCGC	93
TAAA•TTTA	24	TGTT•AACA	51	CGAA•TTCG	70	GGGA•TCCC	106
TTAA•TTAA	24	TTGT•ACAA	51	GTGC•GCAC	71	TGGA•TCCA	106
AATC•GATT	27	CAAA•TTTG	52	ACGC•GCGT	72	CCGA•TCGG	107
GAAT•ATTC	27	CGTT•AACG	52	TTGC•GCAA	72	CGGA•TCCG	107
GTAG•CTAC	27	GAGA•TCTC	53	AGGA•TCCT	73	GGCA•TGCC	109
TAGT•ACTA	27	AGGT•ACCT	55	CAGA•TCTG	73	GGCC•GGCC	109
AGTA•TACT	27	AGTG•CACT	55	CTGA•TCAG	73	GGGC•GCCC	109
CTAA•TTAG	28	CAGT•ACTG	55	GAGG•CCTC	73	TGCA•TGCA	109
TAAG•CTTA	28	CTGT•ACAG	55	GGAG•CTCC	73	TGGC•GCCA	109
TATA•TATA	28	AAGG•CCTT	56	TGAG•CTCA	73	CGCA•TGCG	110
AGAT•ATCT	31	CAAG•CTTG	56	CGAG•CTCG	74	CGGC•GCCG	110
GAAC•GTTC	31	CATA•TATG	56	AGCA•TGCT	76	GCGG•CCGC	110
CTAG•CTAG	32	GAGC•GCTC	56	AGCC•GGCT	76	GGCG•CGCC	110
GAAA•TTTC	32	GGTA•TACC	60	AGGC•GCCT	76	CGCG•CGCG	111
AGAC•GTCT	35	TGTA•TACA	60	CAGC•GCTG	76	GGGG•CCCC	126
AGTC•GACT	35	CGTA•TACG	61	CTGC•GCAG	76	TGGG•CCCA	126
GAGT•ACTC	35	ATGA•TCAT	64	AGCG•CGCT	77	CCGG•CCGG	127
AAGA•TCTT	36	GATG•CATC	64	ATGG•CCAT	84	CGGG•CCCG	127

The TRX scores (**S_TRX_**) of the 136 non redundant tetrameric sequences correspond to the sum of the individual TRX scores (Table 2) of the three complementary dinucleotides composing the tetramers.

Overall, the TRX analysis provides a new and mechanistically based interpretation of the sequence-dependent propensities for wider minor groove in B-DNA. Next, we examine whether DNA binding proteins take advantage of this groove malleability.

### Relevance of DNA intrinsic flexibility for protein binding

To investigate if proteins exploit the intrinsic sequence-dependent malleability of the DNA, we compared the TRX scores of free tetrameric sequences to the minor groove width values recently compiled [6] on 4426 tetramers in crystallographic DNA-protein complexes, and averaged according to the 136 possible tetrameric sequences. In this crystal structure dataset, the proteins bind either the major or minor groove of their DNA targets.

On average, minor groove width values in protein-bound DNA are significantly wider (6.7±1.0 Å, Figure 4-B) than in free DNA (5.8±1.4 Å, Figure 4-A). The minor groove width values of protein-bound DNA parallel well the corresponding TRX scores of intrinsic flexibility for tetrameric sequences (Figure 4-B). This correlation is less dispersed than that obtained with minor groove width in free DNA (Figure 4-A). A possible reason is the larger size of the protein-bound DNA dataset. Also, bound DNA may be less prone to crystal packing biases than free DNA [36].

As shown above, stiff sequences favor narrow minor grooves while free malleable sequences can explore various conformations, from narrow to wide minor grooves. Figure 4 indicates that proteins exploit the ability of flexible tetramers to adopt wide minor grooves. Indeed, the tetramers characterized by higher than average TRX scores (>62) exhibit enlarged minor grooves (width of 7.2±0.6 Å on average) in DNA-protein complexes (Figure 4-B). Most stiffer tetramers (TRX scores <62) maintain narrow minor grooves when bound to proteins (Figure 4-B). However, 24% of stiff tetramers deviate from this general trend, with minor groove widths exceeding 6.7 Å, *i.e.* higher than the overall average. Such cases point out that proteins can sometimes overcome the intrinsic structural preference of DNA.

Also, more specific and sequence-focused arguments reinforce the idea that proteins exploit the intrinsic DNA mechanical properties. In protein-bound DNA, the malleable GC-rich and the stiff AT-rich tetramers are strongly associated to wide and narrow minor grooves, respectively. For instance, the tetramers centered on GpG•CpC, GpC•GpC or CpG•CpG have an average TRX score of 95±21, and an average minor groove width of 7.6±0.6 Å; conversely, the low TRX scores (31±17) of the tetramers centered on ApA•TpT, ApT•ApT and TpA•TpA correspond to narrow minor grooves (5.8±1.0 Å). Still, TpA•TpA is more conductive to BII conformers than ApA•TpT or ApT•ApT (Table 2), and is known to be able to widen the minor groove [43]. The minor groove width of the protein-bound ApTpApT tetramers is plurimodal [6], with some values exceeding 9 Å that do not reflect its low TRX score of 14. This stresses that, although the DNA mechanics is very influential, other factors also contribute to DNA-protein recognition.

Analyzing the observed minor groove width in terms of sequence-dependent intrinsic flexibility provides further insights regarding the dependence on the sequence context. This is well illustrated by the ApA•TpT steps, known to result in narrow minor grooves in both free and bound DNA, especially for “A tracts” [6], [43]. In bound DNA, the average minor groove width of ApA in NpApApN tetramers is 5.6±0.9 Å, below the overall average (6.7±1 Å). In such tetrameric sequences, according to the TRX scores, a substantial flexibility can only come from a 5′-CpA•TpG step, because other possible flanking sequences would provide only modest (TpA•TpA, GpA•TpC) or very low (ApN•NpT) flexibility (Table 2). Actually, among the 16 protein-bound NpApApN tetramers, the widest minor groove width is for ApA in CpApApN, with an average value of 6.4±0.9 Å. A similar effect is observed for ApT•ApT steps. This underlines the importance of a 5′-CpA•TpG neighbor that confers to ApA•TpT and ApT•ApT steps the possibility to accommodate proteins requiring a widened minor groove.

So, our results show significant evidence that the DNA minor-groove deformation upon protein binding is commensurate with the intrinsic nucleotidic sequence-dependent malleability captured by the TRX scale. Under the protein influence, such distortions can be enhanced beyond what is observed in free DNA, but these enhanced deformations still reflect the intrinsic sequence-dependence DNA flexibility.

## Discussion

Analyzing the groove dimensions in DNA-protein complexes highlighted that the DNA minor groove width is especially variable [3], [6], [8], [9]. This raises two main lines of enquiry: i) how can narrow minor grooves accommodate protein elements, and ii) how can minor grooves be widened without a large cost in deformation energy. Progress on the first point was recently achieved by an elegant study showing that the negative electrostatic potential is enhanced in narrow AT-rich minor grooves, which are thus attractive for basic amino acids [6].

Here, we show that the minor and major groove dimensions in DNA are intrinsically coupled to the sequence-dependent BI and BII phosphate group conformations. This coupling occurs *via* the tight correlation existing between these phosphate states and the roll and base-pair displacement parameters [21], [28], [30]–[33]. Importantly, BII-rich segments, characterized by negative rolls and base-pairs displaced towards the major groove, are associated to deeper and widened minor grooves and shallow major grooves.

The experimental TRX scale captures the DNA intrinsic flexibility in terms of backbone conformation, roll, twist and base-pair displacement [19]. In the present work, this scale enables to rationalize and explain why stiff AT-rich and flexible GC-rich regions favor narrow and wide minor grooves, respectively. It uncovered the role of the malleable “mixed” CpA•TpG step in influencing AT-rich regions, by significantly opening their narrow minor grooves. The free DNA propensity to adopt specific groove dimensions is exploited by DNA-binding proteins, as shown by the relation between TRX scores and minor groove width in protein-bound DNA. This relationship can now be seen as broadly general, but it was already incipient in isolated case studies of DNA-protein interactions, both specific and non-specific. First, the transcription factor NF-κB specifically binds the major groove of its targets; these sites contain two conserved BII-rich regions which maintain shallow major grooves so as to expose the base atoms forming specific hydrogen bonds in the complex [22]. Second, the DNase I can interact with any DNA minor groove; yet, preferential cutting sequences are adjacent to BII-rich regions that widen the minor groove and favor binding of this enzyme [13].

Specific and non-specific DNA binding proteins recognize sequence-dependent DNA shapes [5]. The variation of DNA groove dimensions plays a crucial role in such mechanisms. The TRX approach yields a much more precise understanding of the rules linking groove modulation and sequence, and provides a direct mechanistic insight for this phenomenon. Therefore, the present analysis unveiled a new and important guiding principle in the field of DNA-protein interactions.

## Materials and Methods

### Crystallographic datasets

The X-ray structures of free B-DNA include 28 DNA decamers at 2.0 Å resolution or better (list provided in Supporting Information S1). These structures are all double-stranded DNAs, and do not include mismatches or modified bases, sugars or backbones. Three decamers (PDB codes 1SK5, 1ZFG and 1D57) contain several unusual α/γ angles; they were excluded, given i) the rarity of such backbone conformations in free B-DNA in solution [29], [44] and ii) their impact on the helical parameters [28].

### Structure analysis

Analyses of DNA structures were carried out using Curves [45], [46]. The same program was used by Rohs and colleagues [6] who compiled the minor groove widths of a very large dataset of free and bound DNA. Thus, our decamer analysis is consistent with that of Rohs et al.

The phosphate conformations were analyzed in terms of BI and BII states, defined by the values of the two torsion angles ε (C_4′_-C_3′_-O_3′_-P) and ζ (C_3′_-O_3′_-P-O_5′_), with BI corresponding to (ε-ζ)<0° (centered around −90°), and BII corresponding to (ε-ζ)>0° (centered on +90°).

## Supporting Information

Supporting Information S1 Supporting Information S1 contains the list of the free B-DNA X-ray structures of decamers surveyed in this study, and the figure showing the anti-correlations of the minor and major groove widths and depths, respectively, extracted from this X-ray dataset.
(DOC)
