# Supplementary material for: Understanding the Sequence-Dependence of DNA Groove Dimensions: Implications for DNA Interactions
Source: PLoS One. 2010 Dec 29;5(12):e15931. doi: 10.1371/journal.pone.0015931 (PMC3012109; doi:10.1371/journal.pone.0015931)
Supplement: Supporting Information S1 — contains the list of the free B-DNA X-ray structures of decamers surveyed in this study, and the figure showing the anti-correlations of the minor and major groove widths and depths, respectively, extracted from this X-ray dataset. (DOC) [file pone.0015931.s001.doc]

**Table S1**: X-ray structures of free B-DNA surveyed in this study.

| **PDB code** | **NDB code** | **Resolution (Å)** | **sequence** | **N_BII_** |
| --- | --- | --- | --- | --- |
| 1ZFB | bd0081 | 1.65 | CCGCCGGCGG | 9 |
| 3GGB (B) | bd0113 (B) | 0.87 | CCAGGCCTGG | 8 |
| 3GGI (B) | bd0114 (B) | 0.87 | CCAGGCCTGG | 8 |
| 3GGK (B) | bd0115 (B) | 0.87 | CCAGGCCTGG | 8 |
| 1ZF0 | bd0077 | 1.50 | CCGTTAACGG | 7 |
| 1EN8 | bd0034 | 0.95 | CCAACGTTGG | 6 |
| 1EN9 | bd0035 | 0.95 | CCAGCGCTGG | 6 |
| 1ENE | bd0036 | 0.95 | CCAGCGCTGG | 6 |
| 1D8G | bd0023 | 0.74 | CCAGTACTGG | 6 |
| 1BD1 | bdj017 | 1.60 | CCAGGCCTGG | 6 |
| 158D | bdj052 | 1.90 | CCAAGCTTGG | 6 |
| 1EN3 | bd0033 | 0.95 | CCAACGTTGG | 4 |
| 1ZF5 | bd0079 | 0.99 | CCAGCGCTGG | 6 |
| 1ZFF | bd0087 | 0.94 | CCGAATTCGG | 6 |
| 3GGB (A) | bd0113 (A) | 0.87 | CCAGGCCTGG | 6 |
| 3GGI (A) | bd0114 (A) | 0.87 | CCAGGCCTGG | 6 |
| 3GGK (A) | bd0115 (A) | 0.87 | CCAGGCCTGG | 6 |
| 1IKK | bd0051 | 1.60 | CCTTTAAAGG | 4 |
| 5DNB | bdj019 | 1.40 | CCAACGTTGG | 4 |
| 1D23 | bdj025 | 1.50 | CGATCGATCG | 4 |
| 1D49 | bdj031 | 1.50 | CGATTAATCG | 4 |
| 1ZFC | bd0082 | 2.00 | CCGATATCGG | 4 |
| 1D56 | bdj036 | 1.70 | CCAGCGCTGG | 3 |
| 1ZF7 | bd0080 | 1.05 | CCGTCGACGG | 3 |
| [463D](http://www.rcsb.org/pdb/explore/explore.do?structureId=463D) | bd0014 | 1.45 | GCGAATTCGC | 2 |
| [1EHV](http://www.rcsb.org/pdb/explore/explore.do?structureId=1EHV) | bd0032 | 1.80 | GCGAATTCGC | 2 |
| 477D | bd0019 | 1.70 | GCGAATTCGC | 2 |
| 196D | bdj060 | 1.70 | CTCTCGAGAG | 2 |
| n.a. | bdj061 | 1.95 | CCACTAGTGG | 2 |
| 307D | bdj081 | 1.85 | CAAAGAAAAG | 2 |
| 476D | bd0018 | 1.30 | GCGAATTCGC | 1 |
| [1WQY](http://www.rcsb.org/pdb/explore/explore.do?structureId=1WQY) | bd0073 | 2.00 | CCATTAATGG | 1 |

The PDB and NDB codes, the resolution, the sequence and the total number of BII phosphates (N_BII_) are given for each decamer.

**Figure S1**

Relationship between major and minor groove depths and widths.


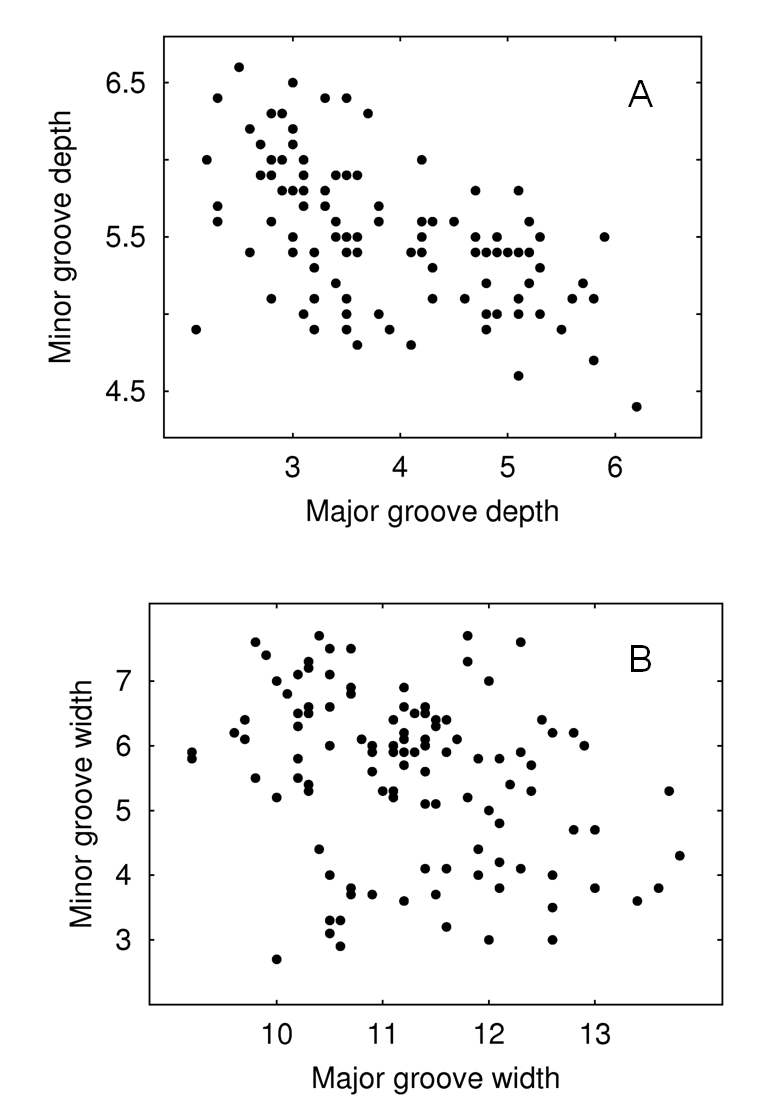


Major and minor groove depths (in Å, panel A) are anti-correlated. Major and minor groove widths (in Å, panel B) follow the same trend, while rather poorly coupled. The major and minor groove depths and widths were extracted from the high resolution X-ray structures of free decamers (Table S1).
